# Biological and clinical stability after switching from intravenous to subcutaneous natalizumab in RRMS under standard and extended dosing

**DOI:** 10.3389/fimmu.2026.1879496

**Published:** 2026-07-22

**Authors:** Alex Agundez-Moreno, Silvia Presas-Rodríguez, Antonio Manuel González-García, Aina Teniente-Serra, Berta Arcos-Ribas, Eva Maria Martínez-Caceres, Luis Querol, Lorena Martín-Aguilar, Ángela Vidal-Jordana, Jose E. Meca Lallana, Camila Dos-Santos, Jorge Millan, Gabriel Valero, Antonio Cano, Virginia Casado, Anuncia Boltes-Alandi, Jose Vicente Hervas-Garcia, Luis Brieva, Gary Alvarez, Cristina Ramo-Tello

**Affiliations:** 1 Department of Immunology, Germans Trias i Pujol Research Institute and University Hospital, Badalona, Spain; 2 Department of Cell Biology, Physiology and Immunology, Universitat Autònoma de Barcelona, Barcelona, Spain; 3 Multiple Sclerosis Unit, Department of Neurosciences, Germans Trias i Pujol University Hospital, Badalona, Spain; 4 Neurology Department, Hospital de la Santa Creu i Sant Pau, IR SANT PAU, Barcelona, Spain; 5 Neurology, Multiple Sclerosis CSUR and Clinical Neuroimmunology Unit, Virgen de la Arrixaca Clinical University Hospital, Cartagena, Spain; 6 Department of Neurology, Hospital de Mataró, Mataró, Spain; 7 Department of Neurology, Hospital de Granollers, Granollers, Spain; 8 Department of Neurology, Hospital Moisès Broggi, Sant Joan Despí, Spain; 9 Department of Neurology, Hospital Arnau Vilanova, Lleida, Spain; 10 Neuroimmunology and Multiple Sclerosis Unit, Neurology Department, Hospital Josep Trueta, Girona, Spain

**Keywords:** CD49d receptor occupancy, extended interval dosing, intravenous administration, lymphocyte subsets, natalizumab, neurofilament light chain (sNfL), relapsing-remitting multiple sclerosis (RRMS), subcutaneous administration

## Abstract

**Introduction:**

Natalizumab (NTZ) is a high-efficacy therapy for relapsing-remitting multiple sclerosis (RRMS) that can be administered intravenously (IV) or subcutaneously (SC). While switching from IV to SC NTZ is increasingly common in clinical practice, evidence on pharmacodynamic stability, particularly under extended interval dosing (6 weeks), remains limited. This study aimed to evaluate CD49d receptor occupancy (RO), serum neurofilament light chain (sNfL) levels, and clinical outcomes in RRMS patients switching from IV to SC NTZ while maintaining either 4- or 6-week dosing intervals.

**Methods:**

We conducted a multicenter, ambispective, observational study including RRMS patients who had received IV NTZ for at least 6 months on a 4w or 6w schedule and subsequently switched to SC NTZ while maintaining the same dosing interval. CD49d RO was assessed in CD4+, CD8+, and CD19+ lymphocyte subsets before the first, third, and seventh SC administrations. sNfL levels and clinical outcomes (relapses, MRI activity, EDSS score) were also evaluated. Mixed models for repeated measures were applied.

**Results:**

A total of 48 patients were included (4w: n=20; 6w: n=28). CD49d RO remained stable over time within both dosing schedules across all lymphocyte subsets. A significant difference between groups was observed only in CD19+ cells at the third administration (LS mean difference 13.08, SE = 4.10; p=0.028; 95% CI: 4.90–21.26) without clinical correlation. sNfL levels showed no significant differences at any timepoint within or between groups. Clinically, patients remained stable, with no new MRI activity or disability progression during the follow-up.

**Conclusion:**

Switching from IV to SC NTZ while maintaining either standard (4w) or extended (6w) dosing intervals preserves pharmacodynamic stability, as reflected by sustained CD49d RO, stable sNfL levels, and consistent clinical outcomes. These findings support SC administration as a reliable alternative to IV NTZ, including in extended interval dosing strategies.

## 1 Introduction

Multiple sclerosis (MS) is a chronic inflammatory and degenerative disease of the central nervous system (CNS) (1) driven by immune-mediated mechanisms that promote lymphocyte migration into the CNS (2). Currently, several disease-modifying therapies (DMTs) are available for patients with relapsing-remitting MS (RRMS), with different routes of administration (3), where treatment decisions increasingly incorporate patient preference and convenience (4).

Natalizumab (NTZ) is a humanized IgG4 monoclonal antibody targeting α4-integrin (CD49d), acting as an allosteric antagonist (5). The vascular cell adhesion molecule-1 (VCAM-1 or CD106), expressed in inflamed cerebrovascular endothelial cells, interacts with very late antigen-4 (VLA-4, α4-β1 integrin), allowing leukocyte migration across the blood-brain barrier (BBB) into the CNS. NTZ binds to VLA-4, inhibiting the adhesion of activated lymphocytes to endothelial cells that express VCAM-1. As a result, NTZ prevents the migration of activated T-cells into the brain and the subsequent inflammatory activity associated with the disease (6).

NTZ, administered intravenously (IV) or subcutaneously (SC) in a 4-week (4w) schedule, is considered one of the most effective therapies for reducing the risk of relapses (7), preventing MS lesion accumulation, and mitigating neurodegeneration, with a selective immunomodulator effect (8, 9). However, its use has been associated with the risk of progressive multifocal leukoencephalopathy (PML), a CNS demyelinating disease caused by John Cunningham (JC) virus reactivation in certain patients (10, 11). As evidenced by previous reports, extended dosing intervals, such as a 6-week (6w) schedule, may reduce PML risk (TOUCH) (12) by partially restoring CNS immune surveillance, without compromising efficacy (13, 14).

The NOVA clinical trial (NCT03689972) (15, 16) evaluated the safety and efficacy of IV NTZ administered every 6w *versus* the standard 4w schedule. The results showed that patients clinically stable on a 4w IV NTZ schedule could safely switch to a 6w interval without loss of efficacy and with potentially lower PML risk. Additionally, the NOVA extension study (17) assessed patient preference for SC *versus* IV administration. Most patients (87.8%) on a 6w schedule preferred SC administration. Safety and efficacy were comparable between routes, and overall disease activity remained low. However, despite the increasing use of SC NTZ and extended dosing strategies in clinical practice, data on CD49d receptor occupancy (RO) following the switch from IV to SC administration, particularly under a 6-week schedule, remain limited.

Therefore, our study aimed to evaluate CD49d RO (8) in lymphocyte subsets in RRMS patients switching from IV to SC NTZ while maintaining either 4- or 6-week dosing intervals. In addition, we assessed serum neurofilament light chain (sNfL) levels and clinical outcomes to provide a comprehensive evaluation of pharmacodynamic stability and disease activity.

## 2 Methods

### 2.1 Study design

**Figure 1:**
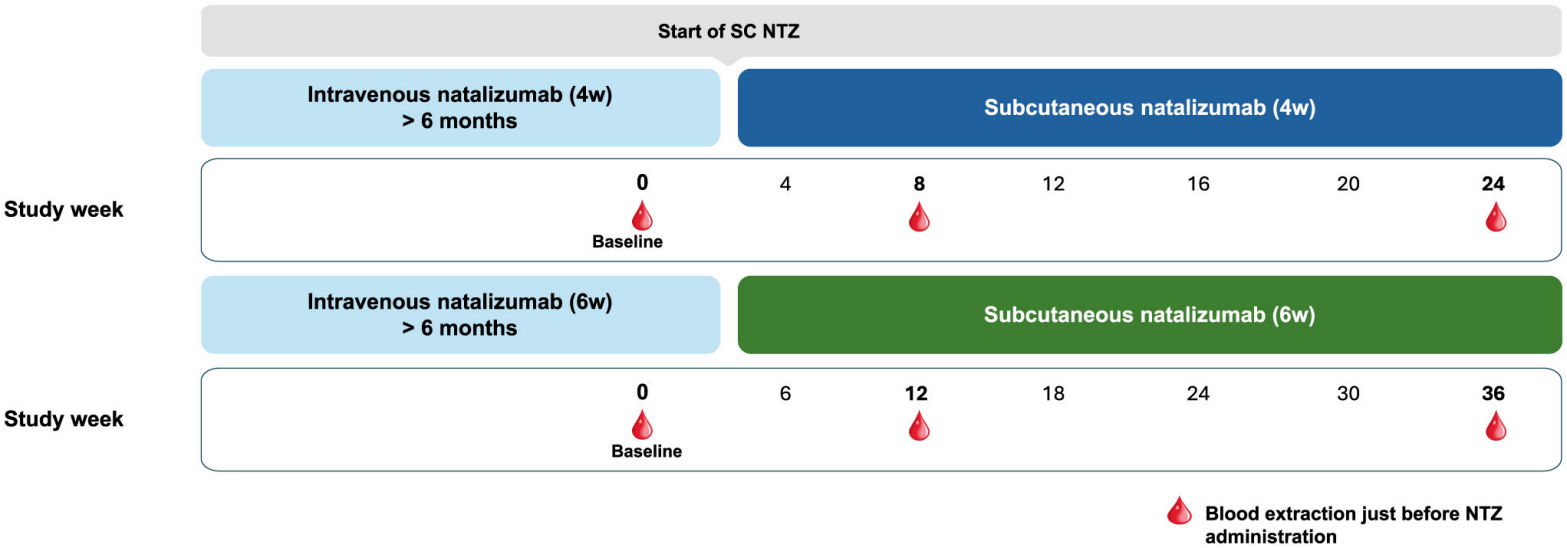
Switch from IV to SC NTZ in RRMS patients. Blood samples were collected at three timepoints: before the first, third and seventh SC administrations. Follow-up lasted 24 weeks in the 4w dosing schedule and 36 weeks for the 6w schedule. IV, intravenous; SC, subcutaneous; NTZ, natalizumab; w: week.

This was a multicenter, observational study with ambispective data collection conducted in RRMS patients between September 2022 and December 2024. This study was approved by the appropriate ethics committees and was performed in the MS Units of 5 hospitals in Catalonia (Spain). Data were collected from patient records according to routine clinical practice. This ambispective cohort study included both retrospective and prospective data collection. Retrospective data were collected from the period before patients switched to SC NTZ. Following the switch, patients in both dosing cohorts (4w and 6w) were prospectively followed, and clinical outcomes were recorded during the follow-up period. A total of 48 adult patients with RRMS were included. In instances where data were unavailable, the exact number of patients included in the analysis is specified. Eligible patients had received IV NTZ every 4w (20 patients) or 6w (28 patients) for at least 6 months prior to switching to SC NTZ. Blood samples were collected at three timepoints: before the first, third, and seventh SC NTZ administrations (**Figure 1**). The follow-up period was 24 weeks for the 4w SC schedule, and 36 weeks for the 6w schedule. Patients were required to have received at least two consecutive SC NTZ administrations during follow-up. The first blood sample, collected before the initial SC NTZ administration dose, served as the baseline (reflecting IV NTZ). The study was conducted and reported in accordance with the STROBE guidelines (18).

### 2.2 Flow cytometry

Whole peripheral blood samples were collected in ethylenediaminetetraacetic acid (EDTA) tubes, stored at room temperature, and processed within 24 hours of collection. Samples were analyzed at the Department of Immunology of Germans Trias i Pujol University Hospital (Badalona, Spain). Three lymphocyte subpopulations (CD4+, CD8+, and CD19+) were evaluated before the first, third and seventh SC NTZ administrations. This study focuses on the analysis of CD4+ T cells, CD8+ T cells, and CD19+ B cells which play key roles in the immunopathogenesis of MS. Studying these subsets provides a representative overview of both major adaptative immune compartments and helps characterize immune activity and treatment response in patients. Quantification of CD49d receptors and bound NTZ molecules was performed by quantitative flow cytometry on T (CD4+ and CD8+) and B (CD19+) lymphocytes.

Calibration and performance tracking of the flow cytometer were conducted prior to sample acquisition with Rainbow 6 Peak calibration particles and QuantiBRITE phycoerythrin (PE) beads (Becton Dickinson, BD). To calibrate the PE fluorescence signal, Rainbow 6 Peak beads were applied, and the photomultiplier tube (PMT) voltage for the fourth brightest PE peak was adjusted to achieve a mean fluorescence intensity (MFI) between 4650 and 4950. A second calibration with QuantiBRITE beads established the correlation between geometric MFI (GeoMFI) and PE molecules on the cell surface.

Briefly, 5 mL of peripheral blood was lysed with a non-fixing, ammonium chloride-based lysing reagent (FACSLysing Solution, BD) for 10 minutes. A total of 1,000,000 cells per tube were incubated for 15 minutes at room temperature with pre-titrated amounts of monoclonal antibodies. The antibodies used were anti-CD3 V450 (clone UCHT1, BD), anti-CD4 FITC (clone SK3, BD), anti-CD8 APC-H7 (clone SK1, BD), anti-CD19 PerCP-Cy5.5 (clone SJ25C1, BD), and either anti-CD49d (clones 9F10, BD) or huIgG4 Fc PE (clone HP6025, Southern Biotech) to measure bound NTZ. After two washes with phosphate-buffered saline (PBS), lymphocytes were acquired using a FACSCanto II flow cytometer (BD), and all samples were analyzed with FACSDiva software (BD) (**Supplementary Figure 1**). In parallel, a Fluorescence Minus-One (FMO) control for PE was included for each sample to set the threshold between negative and positive huIgG4 and CD49d populations.

Surface quantification of huIgG4 and CD49d was performed following the QuantiBRITE manufacturer’s instructions. The number of surface molecules per cell was calculated by linear regression. CD49d RO was expressed as the percentage of NTZ-bound molecules relative to the total CD49d receptor molecules, according to the formula: (bound NTZ molecules/total CD49d molecules) x 100.

### 2.3 sNfL levels

sNfL levels were measured using the Simoa NF-light kit in the SR-X Immunoassay Simoa analyzer (Quanterix Corp., Boston, MA). Samples were analyzed according to the manufacturer’s instructions and standard procedures. All sNfL values were within the assay linear range. The intra-assay and inter-assay coefficients of variation (CVs) at an intermediate concentration level were 5.7% and 14.5%, respectively.

### 2.4 Clinical assessments

Baseline data included demographic characteristics, disease duration, prior disease-modifying therapies, JC virus serology, duration of previous IV NTZ treatment, annualized relapse rate (ARR) in the last year, prior magnetic resonance imaging (MRI) activity, and scores for the Expanded Disability Status Scale (EDSS).

The ARR was calculated as the number of relapses occurring during the 12 months prior to the treatment switch. A relapse was defined as the acute or subacute onset of new neurological symptoms, or worsening of pre-existing symptoms, lasting at least 24 hours in the absence of fever or infection, and accompanied by objective neurological findings.

Patients were monitored for 24 weeks and 36 weeks in the 4w and 6w dosing schedules, respectively. The clinical course was evaluated using the occurrence of relapses and the EDSS scores following the switch from IV to SC NTZ. Radiological activity was assessed based on the appearance of new lesions on brain MRI acquired during follow-up, compared with the previous MRI. The available scans, obtained according to clinical practice, included T1, T2, T2/FLAIR, T1 post-gadolinium and susceptibility-weighted sequences. No spinal cord MRI was evaluated.

### 2.5 Statistical analysis

Mixed models for repeated measures (MMRMs) were used to study the evolution over time and by therapeutic schedule of the variables CD49d RO (CD4+, CD8+ and CD19+) and sNfL. An unstructured covariance matrix was specified to model within-subject correlations over time, and p-value multiplicity adjustment for multiple comparisons across therapeutic schedules and timepoints was performed using the Tukey method. No explicit imputation methods were applied to handle missing data. The software used for the MMRM analyses was SAS version 9.4, under Enterprise Guide Interface v8.3. The least squares (LS) means and LS mean differences between therapeutic schedules, along with their 95% confidence intervals (CIs), were estimated. Clinical data were prospectively collected using the REDCap (Research Electronic Data Capture) platform, a secure web-based application for research data management. Demographic and clinical characteristics were summarized using descriptive statistics (number of patients with data, mean, standard deviation [SD], percentage). Additional descriptive analyses were performed using IBM SPSS Statistics software.

## 3 Results

### 3.1 Demographic and clinical characteristics

Fifty patients with RRMS who switched from IV NTZ to SC NTZ were recruited. Of these, two were excluded because they were on a different dosing schedule of NTZ (5 weeks), so a total of 48 patients with RRMS who switched from IV NTZ to SC NTZ were included in the study. Patients were classified into two groups based on their prior IV dosing schedule. A total of 20 patients received NTZ every 4w (4w group), and 28 patients every 6w (6w group). Baseline demographic characteristics were broadly similar between groups. However, differences in prior NTZ exposure and pre-switch disease activity were observed, with patients in the 4W cohort showing shorter treatment duration, higher ARR, and more radiological activity, as they had switched to SC NTZ within less than one year of initiating IV NTZ. Radiological activity observed in some patients on the baseline MRI (prior to SC NTZ initiation) reflects disease activity that occurred before IV NTZ initiation, as these patients switched to SC NTZ within less than one year of starting IV NTZ and had no interim MRI available. The demographic and clinical characteristics of both groups before switching to SC NTZ are summarized in **Table 1**.

**Table 1:** Baseline characteristics of patients with RRMS.

Variable	Overall (N = 48)	4w (N = 20)	6w (N = 28)
Females, n (%)	34 (70.8)	13 (76.5)	21 (75)
Age (years), median (IQR)	42 (37.8-50)	41 (36.8-45.5)	46.5 (40.3-50.8)
BMI, mean (SD)	25.3 (4.2)	25.6 (4.9)	24.9 (3.8)
Disease duration (years), mean (SD)	11.7 (6.8)	9.5 (5.5)	13.2 (7.4)
Patients previously treated with DMTs, n (%)	39 (81.3)	15 (75)	24 (85.7)
Platform agents #	29	10	19
Fumarates	9	6	3
SP1	6	3	3
Alemtuzumab Cladribine	1 1	0 1	1 0
Positive JCV serology, n (%)	9 (19.7)	2 (10)	7 (25)
Previous IV-NTZ duration (years), median (IQR)	3.5 (1-9)	1 (0-5)	5.5 (3-10.5)
ARR last year, mean (SD)	0.15 (0.3)	0.4 (0.65)	0
Radiological activity (last MRI)*, n (%)	8 (16.6)	8 (40)	0
EDSS, mean (SD)	2.5 (1.7)	2 (1.4)	2.5 (1.7)

Baseline is considered the first blood sample collected before the initial SC NTZ administration dose (reflecting IV NTZ).

IQR, interquartile range; SD, standard deviation; BMI, body mass index; DMT, disease-modifying therapy; SP1, sphingosine 1-phosphate receptor modulators; JCV, John Cunningham virus; IV, intravenous; SC, subcutaneous; NTZ, natalizumab; ARR, annualized relapse rate; MRI, magnetic resonance imaging; EDSS, expanded disability status scale. ^#^Platform agents, interferons and glatiramer acetate. *MRI activity prior to the initiation of NTZ treatment.

### 3.2 Monitoring CD49d RO

**Figure 2:**
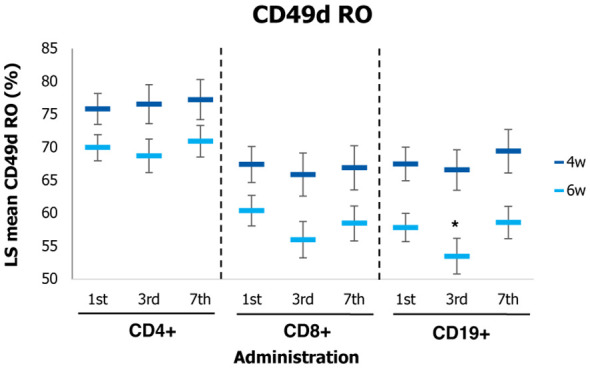
CD49d RO in CD4+, CD8+ and CD19+ lymphocyte subpopulations in SC NTZ under 4w and 6w dosing schedules (by group). The number of patients included at each timepoint was: first administration (4w: n=20; 6w: n=28), third administration (4w: n=18; 6w: n=25), and seventh administration (4w: n=17; 6w: n=28). CI, confidence interval; LS, least squares; RO, receptor occupancy; w: week. Estimates were obtained using an MMRM to evaluate changes over time and between groups. *p*-values for LS mean difference have been computed using the Tukey adjust method (*p-value ≤0.05).

CD49d RO percentage (**Figure 2**) remained stable over time following the switch to SC NTZ in both the 4w (N = 20) and 6w (N = 28) dosing schedules across all lymphocyte subpopulations (CD4+, CD8+ and CD19+) at the evaluated timepoints (first, third and seventh administrations; **Table 2**) in both the 4w (N = 20) and 6w (N = 28) groups. No statistically significant changes from baseline were observed within groups in any subpopulation (**Tables 3**–**5**). A statistically significant difference between groups was identified only in CD19+ cells prior the third administration (LS mean difference 13.08; 95% CI 4.90-21.26); p = 0.028).

**Table 2:** Monitoring CD49d RO in CD4+, CD8+ and CD19+ lymphocyte subpopulations in SC NTZ under 4w (N  =  20) and 6w (N  =  28) dosing schedules.

CD49d RO %	Mean (LS)	Standard error (LS)	95% CI	p-value
CD4+ in the 4w schedule				0.843
First administration	75.62	2.50	(70.40; 80.85)	
Third administration	76.60	2.95	(70.43; 82.77)	
Seventh administration	77.00	3.15	(70.41; 83.59)	
CD4+ in the 6w schedule				0.566
First administration	69.99	1.86	(66.18; 73.81)	
Third administration	68.69	2.27	(64.03; 73.35)	
Seventh administration	70.86	2.52	(65.68; 76.04)	
CD8+ in the 4w schedule				0.855
First administration	67.43	3.45	(60.22; 74.64)	
Third administration	65.80	3.07	(59.37; 72.22)	
Seventh administration	66.20	3.72	(58.42; 73.98)	
CD8+ in the 6w schedule				0.187
First administration	60.37	1.79	(56.71; 64.04)	
Third administration	55.81	2.32	(51.06; 60.56)	
Seventh administration	58.49	2.86	(52.62; 64.36)	
CD19+ in the 4w schedule				0.652
First administration	67.23	2.82	(61.32; 73.13)	
Third administration	66.31	2.89	(60.25; 72.37)	
Seventh administration	69.36	3.05	(62.97; 75.75)	
CD19+ in the 6w schedule				0.067
First administration	58.03	1.94	(54.06; 62.00)	
Third administration	53.39	2.28	(48.72; 58.06)	
Seventh administration	58.66	2.90	(52.71; 64.61)	

Estimations were obtained using an MMRM to assess changes in CD49d RO over time.

RO, receptor occupancy; MMRM, mixed model for repeated measures; LS,, least squares; CI, confidence interval; w, week; N, number of patients included in the group.

**Table 3:** Monitoring of CD49d RO in CD4+ lymphocyte subpopulation under SC NTZ dosing schedules (4w and 6w).

CD49d RO %	4w (N = 20)	6w (N = 28)	p-value
Mixed model for repeated measures: CD4+			< 0.001
Model fixed effects			
group			0.036
administration			0.638
group*administration			0.825
First administration			
n	20	28	
LS mean (SE)	75.83 (2.33)	69.95 (1.97)	
LS mean 95% CI	(71.14; 80.52)	(65.99; 73.92)	
LS mean difference (SE)	5.87 (3.05)		0.399
LS mean difference 95% CI	(-0.27; 12.02)		
Third administration			
n	18	25	
LS mean (SE)	76.56 (2.95)	68.70 (2.54)	
LS mean 95% CI	(70.62; 82.51)	(63.59; 73.82)	
LS mean difference (SE)	7.86 (3.89)		0.346
LS mean difference 95% CI	(0.08; 15.64)		
Seventh administration			
n	17	28	
LS mean (SE)	77.25 (3.05)	70.91 (2.41)	
LS mean 95% CI	(71.11; 83.40)	(66.05; 75.77)	
LS mean difference (SE)	6.34 (3.89)		0.583
LS mean difference 95% CI	(-1.44; 14.12)		

Estimates were obtained using an MMRM to evaluate changes over time and between groups.

RO, receptor occupancy; MMRM, mixed model for repeated measures; LS, least squares; SE, standard error; CI, confidence interval; w, week; N, number of patients included in the group; n, number of patients with data available for the corresponding analysis.

**Table 4:** Monitoring CD49d RO in CD8+ lymphocyte subpopulation under SC NTZ dosing schedules (4w and 6w).

CD49d RO %	4w (N = 20)	6w (N = 28)	p-value
Mixed model for repeated measures: CD8+			< 0.001
Model fixed effects			
group			0.014
administration			0.368
group*administration			0.797
First administration			
n	20	28	
LS mean (SE)	67.41 (2.75)	60.38 (2.32)	
LS mean 95% CI	(61.88; 72.94)	(55.71; 65.06)	
LS mean difference (SE)	7.03 (3.60)		0.383
LS mean difference 95% CI	(-0.22; 14.27)		
Third administration			
n	18	25	
LS mean (SE)	65.86 (3.27)	55.98 (2.78)	
LS mean 95% CI	(59.28; 72.44)	(50.38; 61.58)	
LS mean difference (SE)	9.88 (4.29)		0.211
LS mean difference 95% CI	(1.31; 18.44)		
Seventh administration			
n	17	28	
LS mean (SE)	66.92 (3.36)	58.45 (2.66)	
LS mean 95% CI	(60.16; 73.68)	(53.10; 63.81)	
LS mean difference (SE)	8.47 (4.28)		0.369
LS mean difference 95% CI	(-0.09; 17.02)		

Estimates were obtained using an MMRM to assess changes in CD49d RO over time and between groups.

RO, receptor occupancy; MMRM, mixed model for repeated measures; LS, least squares; SE, standard error; CI, confidence interval; w,: week; N, number of patients included in the group; n, number of patients with data available for the corresponding analysis.

**Table 5:** Monitoring CD49d RO in CD19+ lymphocyte subpopulation under SC NTZ dosing schedules (4w and 6w).

CD49d RO %	4w (N = 20)	6w (N = 28)	p-value
Mixed model for repeated measures: CD19+			0.0075
Model fixed effects			
group			< 0.001
administration			0.219
group*administration			0.741
First administration			
n	20	28	
LS mean (SE)	67.48 (2.53)	57.82 (2.14)	
LS mean 95% CI	(62.40; 72.57)	(53.52; 62.13)	
LS mean difference (SE)	9.66 (3.31)		0.055
LS mean difference 95% CI	(3.00; 16.32)		
Third administration			
n	18	25	
LS mean (SE)	66.55 (3.07)	53.47 (2.72)	
LS mean 95% CI	(60.36; 72.74)	(47.99; 58.96)	
LS mean difference (SE)	13.08 (4.10)		0.028
LS mean difference 95% CI	(4.90; 21.26)		
Seventh administration			
n	17	28	
LS mean (SE)	69.40 (3.29)	58.59 (2.47)	
LS mean 95% CI	(62.77; 76.03)	(53.61; 63.57)	
LS mean difference (SE)	10.81 (4.12)		0.109
LS mean difference 95% CI	(2.60; 19.02)		

Estimates were obtained using an MMRM to assess changes in CD49d RO over time and between groups.

RO, receptor occupancy; MMRM, mixed model for repeated measures; LS, least squares; SE, standard error; CI, confidence interval; w, week; N number of patients included in the group; n, number of patients with data available for the corresponding analysis.

### 3.3 Clinical data

Of the 48 patients included, three discontinued during the study: two returned to IV NTZ due to adverse events attributed to the SC formulation, and one switched to another treatment because of an increase in the JC virus serology index during follow-up. Among the remaining patients (N = 45), both clinical and radiological outcomes remained stable throughout follow-up.

Regarding radiological activity, four patients showed an increase in the number of brain MRI lesions, but none of these lesions showed gadolinium enhancement (Gd+). In fact, in two patients this finding reflected activity relative to the baseline MRI performed prior to IV NTZ, as they had switched to SC NTZ within less than one year of initiating IV NTZ.

Only one patient in the 4w group experienced a mild sensory relapse after the third SC NTZ administration, associated with a 0.5-point increase in EDSS. However, brain MRI revealed no new or enlarging lesions or signs of inflammatory activity, and treatment was continued without further complications. No other relapses or disability progression was observed during follow-up (**Table 6**).

**Table 6:** Clinical course and MRI in 4w and 6w dosing schedules after a follow-up of 24 weeks and 36 weeks, respectively.

Variable	Overall (N = 45)	4w (N = 17)	6w (N = 28)
Relapses, n (%)	1 (2.2)	1 (5.8)	0 (0)
EDSS, mean (SD)	2 (1.5)	2 (1.2)	2 (1.6)
MRI activity (new lesions), n (%)	4 (8.9)	3 (17.6)	1 (3.8)

EDSS, Expanded Disability Status Scale; SD, standard deviation; MRI, magnetic resonance imaging; N, number of patients included in the group; n, number of patients with data available for the corresponding analysis.

### 3.4 Monitoring sNfL levels

**Figure 3:**
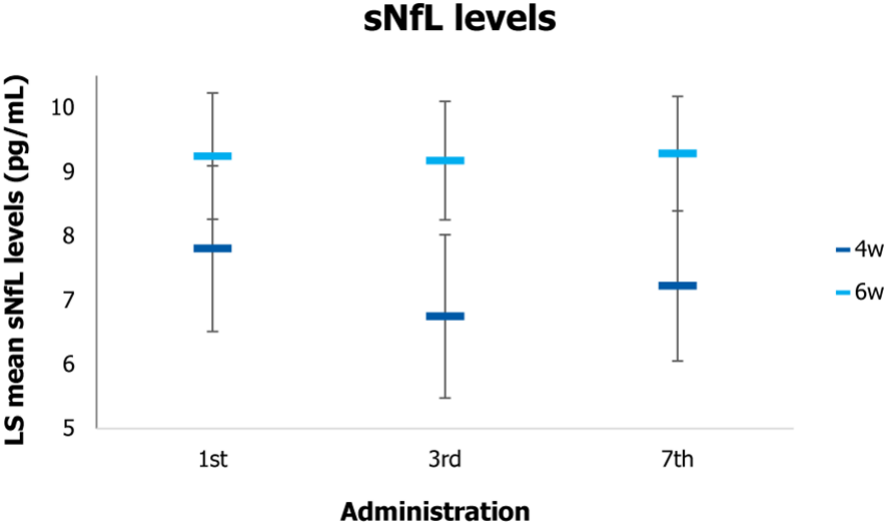
sNfL levels in SC NTZ dosing schedules 4w and 6w (by group). The number of patients included at each timepoint was: first administration (4w: n=16; 6w: n=27), third administration (4w: n=11; 6w: n=24), and seventh administration (4w: n=16; 6w: n=28). LS, least squares; sNfL, serum neurofilament light chain; w, week. Estimates were based on an MMRM evaluating the evolution of sNfL over time and by group. *p*-values for LS mean difference have been computed using the Tukey adjust method.

sNfL levels (**Figure 3**) remained stable over time following SC NTZ administration in both the 4w (N = 16) or 6w (N = 28) groups (**Table 7**). No statistically significant changes were observed within groups across timepoints, nor were there significant differences between groups compared to baseline (**Table 8**).

**Table 7:** Monitoring sNfL levels in SC NTZ dosing schedules 4w (N = 16) and 6w (N = 28).

sNfL levels	Mean (LS)	Standard error (LS)	95% CI	p-value
4w schedule				0.564
First administration	7.81	0.94	(5.81; 9.81)	
Third administration	6.85	0.44	(5.90; 7.80)	
Seventh administration	7.23	0.91	(5.30; 9.17)	
6w schedule				0.982
First administration	9.23	1.10	(6.98; 11.49)	
Third administration	9.14	1.07	(6.95; 11.33)	
Seventh administration	9.27	0.98	(7.25; 11.29)	

Estimates are based on an MMRM evaluating the evolution of sNfL over time.

sNfL, serum neurofilament light chain; MMRM, mixed model for repeated measures; LS, least squares; CI, confidence interval; w, week; N, number of patients included in the group.

**Table 8:** Monitoring sNfL levels in SC NTZ dosing schedules (4w and 6w).

sNfL levels	4w (N = 16)	6w (N = 28)	p-value
Mixed model for repeated measures: sNfL			< 0.001
Model fixed effects			
group			0.163
administration			0.623
group*administration			0.685
First administration			
n	16	27	
LS mean (SE)	7.81 (1.29)	9.25 (0.98)	
LS mean 95% CI	(5.21; 10.41)	(7.27; 11.23)	
LS mean difference (SE)	-1.44 (1.62)		0.947
LS mean difference 95% CI	(-4.71; 1.83)		
Third administration			
n	11	24	
LS mean (SE)	6.75 (1.27)	9.18 (0.92)	
LS mean 95% CI	(4.20; 9.31)	(7.33; 11.02)	
LS mean difference (SE)	-2.43 (1.56)		0.635
LS mean difference 95% CI	(-5.58; 0.73)		
Seventh administration			
n	16	28	
LS mean (SE)	7.23 (1.17)	9.29 (0.89)	
LS mean 95% CI	(4.87; 9.60)	(7.49; 11.09)	
LS mean difference (SE)	-2.06 (1.47)		0.727
LS mean difference 95% CI	(-5.03; 0.91)		

Estimates are based on an MMRM assessing the evolution of sNfL levels over time and by group.

sNfL, serum neurofilament light chain; MMRM, mixed model for repeated measures; LS, least squares; SE, standard error; Cl, confidence interval; w, week; N, number of patients included in the group; n, number of patients with data available for the corresponding analysis.

## 4 Discussion

The development of disease-modifying therapies such as NTZ, one of the most effective therapies for RRMS (7), has significantly improved the management of patients with RRMS, although its use is limited by the risk of PML (19). Extending the dosing interval of NTZ from 4w to 6w has demonstrated promising results in reducing the risk of PML in both Spanish national (20) and international studies (14, 21). In addition, SC administration has been shown to be safe and is preferred by patients, as reported in the NOVA extension study (17). Remarkably, by 30 June 2023, over 21,400 patients had been treated with SC NTZ (22). In this context, NTZ holds a well-established place as a DMT in patients with RRMS, as recognized in a Spanish consensus (23), but also in international guidelines (24, 25). Nonetheless, evidence regarding safety, pharmacodynamic and efficacy under extended interval dosing remains limited.

In this exploratory real-world multicenter study, switching from IV to SC NTZ while maintaining either standard (4w) or extended (6w) dosing intervals preserved CD49d RO, sNfL levels, and clinical stability over time. These findings may support the pharmacodynamic and clinical reliability of SC NTZ administration across dosing regimens, including extended interval strategies. In this context, our results demonstrate that CD49d RO remained stable following the switch from IV to SC NTZ in both dosing schedules across all lymphocyte subpopulations analyzed. These findings are consistent with previous studies indicating that disease activity in MS is closely related to the degree of α4-integrin receptor saturation on lymphocytes (26, 27).

Importantly, the observed stability in RO suggests that SC administration maintains the pharmacodynamic effect of NTZ after switching from IV treatment. This reinforces the value of CD49d RO as a robust pharmacodynamic biomarker and supports its use to monitor treatment stability in clinical practice. Notably, pharmacodynamic stability was maintained not only under the standard 4-week regimen but also in the extended 6-week dosing schedule, where available evidence remains limited. Although lower levels of receptor occupancy have been reported with extended interval dosing in different lymphocyte subsets, this did not translate into increased disease activity in our cohort, in line with previous reports (16, 27). This pattern may reflect a shift in immune engagement and restoration of CNS immune surveillance without apparent loss of disease control and mitigation of PML risk. These findings further support the feasibility of extended interval dosing strategies, even after switching to SC administration.

Regarding clinical outcomes, no meaningful disease activity was observed during follow-up. Only one patient experienced a mild relapse, and a small number of patients showed new MRI lesions without gadolinium enhancement, none of which were associated with clinical deterioration. Overall, patients remained clinically and radiologically stable, supporting the effectiveness of SC NTZ in maintaining disease control. Similarly, sNfL levels remained stable over time in both dosing groups, with no significant differences observed either within or between groups. Given that sNfL is a well-established biomarker of neuroaxonal damage, its stability further supports the absence of ongoing inflammatory or neurodegenerative activity in this cohort and aligns with the observed clinical outcomes.

Beyond efficacy and safety, SC administration offers practical advantages, including increased convenience and patient preference, as highlighted in the NOVA extension study (17). Additionally, SC NTZ has been associated with reduced healthcare resource utilization and lower costs compared to IV administration (28). These aspects are particularly relevant in the context of long-term disease management.

This study has several limitations. The relatively small sample size may limit the statistical power to detect subtle differences between groups, particularly for infrequent clinical outcomes such as relapses or MRI activity. Therefore, the absence of statistically significant differences should not be interpreted as formal evidence of equivalence between dosing schedules or routes of administration. In addition, follow-up duration may not fully capture long-term clinical and safety outcomes. MRI assessments were not systematically performed at predefined intervals, which may reduce sensitivity for detecting subclinical disease activity. Although demographic characteristics were broadly similar between groups, patients receiving the 6w schedule had longer prior NTZ exposure and lower pre-switch disease activity. These differences likely reflect clinical selection for extended interval dosing in routine practice and should be considered when interpreting between-group comparisons.

Body weight and BMI may influence natalizumab pharmacokinetics and RO, particularly under extended interval dosing. However, the limited sample size and the very low number of patients with clinical or radiological activity precluded robust evaluation of this relationship. Exploratory analyses conducted in a small subset of patients did not yield interpretable results. Therefore, the potential impact of anthropometric factors on pharmacodynamic outcomes could not be adequately assessed and should be explored in future studies.

Overall, the real-world multicenter design of this study provides valuable insights into routine clinical practice and supports the generalizability of the findings.

### 4.1 Conclusions

Switching from IV to SC NTZ while maintaining either standard (4w) or extended (6w) dosing intervals preserves pharmacodynamic stability, as reflected by sustained CD49d RO, stable sNfL levels, and consistent clinical outcomes in patients with RRMS. These findings may support SC administration as a reliable and convenient alternative to IV infusion, including in extended interval dosing strategies. Furthermore, CD49d RO may represent a valuable tool for monitoring treatment response and supporting individualized therapeutic approaches in clinical practice. However, confirmation in larger cohorts with longer follow-up is warranted.

## Data Availability

The original contributions presented in the study are included in the article/**Supplementary Material**. Further inquiries can be directed to the corresponding author.
