# Supplementary material for: Biological and clinical stability after switching from intravenous to subcutaneous natalizumab in RRMS under standard and extended dosing
Source: Front Immunol. 2026 Jul 22;17:1879496. doi: 10.3389/fimmu.2026.1879496 (PMC13437448; doi:10.3389/fimmu.2026.1879496)
Supplement: Supplementary file 1 [file SupplementaryFile1.docx]

Supplementary Material

# 1 Supplementary Figure

**Supplementary Figure 1. Flow cytometry analysis of CD49d RO in three lymphocyte subpopulations: CD4+, CD8+ and CD19+.** RO: receptor occupancy.
